# Smoking-associated risks of conventional adenomas and serrated polyps in the colorectum

**DOI:** 10.1007/s10552-014-0513-0

**Published:** 2014-12-24

**Authors:** Jane C. Figueiredo, Seth D. Crockett, Dale C. Snover, Carolyn B. Morris, Gail McKeown-Eyssen, Robert S. Sandler, Dennis J. Ahnen, Douglas J. Robertson, Carol A. Burke, Robert S. Bresalier, James M. Church, Timothy R. Church, John A. Baron

**Affiliations:** 1 Department of Preventive Medicine, Keck School of Medicine, University of Southern California, Los Angeles, CA USA; 2 Division of Gastroenterology and Hepatology, University of North Carolina School of Medicine, Chapel Hill, NC USA; 3 Department of Laboratory Medicine and Pathology, University of Minnesota Medical School, Minneapolis, MN USA; 4 Dalla Lana School of Public Health, University of Toronto, Toronto, ON Canada; 5 Division of Gastroenterology, Department of Veterans Affairs Eastern Colorado Health Care System, University of Colorado School of Medicine, Denver, CO USA; 6 White River Junction VA Medical Center, Geisel School of Medicine at Dartmouth, Hanover, NH USA; 7 Department of Gastroenterology and Hepatology, Cleveland Clinic, Cleveland, OH USA; 8 Department of Gastroenterology, The University of Texas MD Anderson Cancer Center, Houston, TX USA; 9 Department of Colorectal Surgery, Cleveland Clinic, Cleveland, OH USA; 10 Division of Environmental Health Sciences, School of Public Health, University of Minnesota, Minneapolis, MN USA

**Keywords:** Smoking, Tobacco, Colorectal, Adenomas, Serrated polyps

## Abstract

**Purpose:**

Prior studies suggest cigarette smoking is associated with 1.5- to twofold increased risk of colorectal adenomas and possibly a higher risk of serrated polyps. Further clarification of risk differences between adenomas and serrated polyps is needed with regard to co-occurrence and polyp location.

**Methods:**

We conducted a combined analysis of conventional adenoma and serrated polyp occurrence using individual-level data from 2,915 patients participating in three colonoscopy-based clinical trials. All participants had ≥1 adenomas removed at baseline and were followed for up to 4 years. Smoking habits and other lifestyle factors were collected at baseline using questionnaires. We used generalized linear regression to estimate risk ratios and 95 % confidence intervals.

**Results:**

Smokers were at slightly increased risk of adenomas compared to never smokers [current: RR 1.29 (95 % CI 1.11–1.49) and former: RR 1.18 (1.05–1.32)]. Smoking was associated with greater risk of serrated polyps [current: RR 2.01 (1.66–2.44); former: RR 1.42 (1.20–1.68)], particularly in the left colorectum. Associations between current smoking and occurrence of serrated polyps only [RR 2.33 (1.76–3.07)] and both adenomas and serrated polyps [RR 2.27 (1.68–3.06)] were more pronounced than for adenomas only [RR 1.31 (1.08–1.58)]. Results were similar for other smoking variables and did not differ by gender or for advanced adenomas.

**Conclusions:**

Cigarette smoking has only a weak association with adenomas, but is associated with a significantly increased risk of serrated polyps, particularly in the left colorectum. Since a minority of left-sided serrated polyps is thought to have malignant potential, the role of smoking in initiation phases of carcinogenesis is uncertain.

## Introduction

Cigarette smoking is a known risk factor for cancer in several organs, including those not directly exposed to inhaled smoke [1]. In the colorectum, smoking has been inconsistently associated with the development of cancer, but recent meta-analyses showed a moderate increase in risk [2–4]. Studies examining conventional adenomas (also called “adenomatous polyps” or “adenomas”), the precursor lesions to the majority of colorectal cancers (CRC), have observed a more consistent association with smoking [5]. A recent meta-analysis found that current smokers had a twofold elevation in risk of conventional adenomas compared to never smokers (OR 2.14; 95 % CI 1.86–2.46), with a more pronounced association with advanced adenomas [5]. Some [6, 7] but not all [8, 9] studies have suggested that smoking is associated with a higher risk of adenomas occurring in the left colorectum compared to the right colon. Several other studies have also identified duration of tobacco use as a relevant factor [10–15].

It is now well recognized that conventional adenomas are not the sole precursor lesions for CRC. The family of serrated polyps includes hyperplastic polyps (HPs), which are not thought to have significant malignant potential, and two premalignant lesions, sessile serrated adenomas/polyps (SSA/Ps) and traditional serrated adenomas (TSAs), which give rise to up to one-third of sporadic CRC [16]. HPs, SSA/Ps, and TSAs represent roughly 85, 15, and 1 % of serrated polyps, respectively [17]. In contrast to conventional adenomas, there is limited understanding of the risk factors and natural history of premalignant serrated polyps [18]. A few epidemiologic studies have reported associations between smoking and the development of serrated polyps in general with some inconsistencies [9–12, 19]. Morimoto et al. found that smoking was associated with an increased risk of serrated polyps, but not of adenomas [10]. Similarly, two other investigations also reported that smoking was associated with a greater risk of serrated polyps than adenomas [6, 19]. All three studies [6, 10, 19] found that the smoking-associated risk was highest for co-occurrence of adenomas and serrated polyps. This pattern of findings has led to the hypothesis that the co-occurrence of both adenomas and serrated polyps may represent a specific phenotype, which could explain the apparent associations of smoking with risk of adenomas that has been reported in other studies [10]. However, another recent study did not show evidence of a separate phenotype [20], shedding doubt on this phenomenon. Here, we expand upon our findings [8, 21] to report the combined results from three clinical trials with a total of 2,915 participants regarding the associations of recency, intensity, pack-years, duration, and timing of smoking with risk of conventional colorectal adenomas and serrated polyps considering size, concurrence, location, and histology.

## Methods

### Study design

We conducted an analysis using individual-level data from three clinical trials conducted by the Polyp Prevention Study Group In brief, all three trials were randomized, double-blind, placebo-controlled trials to test the efficacy of selected agents for the prevention of colorectal adenomas in individuals with a personal history of these polyps. In the Antioxidant Polyp Prevention Study (APPS), 864 individuals were randomized to beta-carotene (25 mg daily), vitamin C (1 g daily), vitamin E (400 mg daily), beta-carotene plus vitamins C and E, or placebo [8, 22]. Similarly, the Calcium Polyp Prevention Study (CPPS) had 930 subjects were randomized to calcium carbonate [3 g (1,200 mg of elemental calcium) daily] or placebo [23]. In AFPPS (the Aspirin/Folic Acid Polyp Prevention Study), 1,121 subjects were randomized to one of three aspirin groups (placebo, 81 or 321 mg/day) and to one of two folic acid groups (placebo or 1 mg/day) [24]. Follow-up colonoscopic examinations were expected at 3 years after the baseline examination. No individual participated in more than one trial.

### Questionnaires—smoking and other risk factors

All participants in these three trials completed similar questionnaires regarding personal characteristics, medical history, and lifestyle habits. Cigarette smoking status was assessed at study entry through a questionnaire. Current smokers responded as to the number of cigarettes smoked daily, current and former smokers indicated the age of initiation of smoking and the most cigarettes they routinely smoked a day for at least a year, and former smokers indicated the age at smoking cessation and the number of cigarettes per day they formerly smoked.

### Study outcomes

Polyp occurrence in all studies was determined by colonoscopy. Records for all large bowel procedures (endoscopy or surgery) were obtained. Slides for all tissue removed from the bowel after the baseline exam were obtained and sent to a single study pathologist (DCS) for uniform review. Polyps were classified as conventional adenomas or serrated lesions, which included sessile serrated adenoma/polyps, traditional serrated adenomas, and HPs. Since these studies were completed before the current nomenclature regarding classification of serrated polyps was developed, all serrated polyps of any type were analyzed as a group. Large serrated polyps were defined as lesions equal to or greater than 1 cm in estimated diameter, as assessed by the endoscopist. Adenomas were subclassified as tubular, tubulovillous, or villous. Advanced adenomas were considered lesions with any villous histology, size greater than or equal to 1 cm, or high-grade dysplasia/cancer as previously described by the Polyp Prevention Group [8, 22, 23] and others [25]. Serrated polyps were considered as a separate end point. Polyps occurring in the cecum, ileum, ascending and transverse colon were considered right-sided, and lesions occurring in the splenic flexure, descending colon, sigmoid colon or rectum were considered left-sided. For APPS and CPPS, we included findings occurring on exams after the year one colonoscopy up to and including the year four surveillance exam. For AFPPS, we included findings more than 1 year after randomization up to and including the year three surveillance exam.

### Statistical Methods

Chi-square and Student’s *t* tests (for categorical and continuous variables, respectively) were used to compare demographics and baseline characteristics of participants in the pooled trials. Generalized linear models were used to compute crude and adjusted risk ratios (RR) of the association between smoking and risk of each of the precursor lesions of interest. Linear trends were assessed between smoking groups per end point by contrasting parameter estimates calculated from the models. The end points of interest were as follows: (a) one or more conventional adenoma (irrespective of whether there was a serrated lesion present); (b) one or more serrated polyps (irrespective of conventional adenomas); and (c) concurrent conventional adenomas and serrated lesions. RR and 95 % confidence intervals (CI) were adjusted for age, sex, clinical center, and trial. Additionally, the RR of outcomes of conventional adenoma only, serrated polyps only, and concurrent conventional adenomas and serrated polyps were compared side-by-side using a common control group to compare RR and 95 % CI associated with conventional adenomas only, serrated only, and conventional adenomas and serrated polyps jointly.

The possibility that gender modified the smoking associations was assessed in these models with the use of interaction terms and Wald tests (with and without adjustment for other variables). We also assessed associations for polyps by location in the bowel and (for adenomas) advanced features. To ensure associations between smoking variables and risk did not differ by treatment groups in the individual trials, we examined these associations within placebo and treatment groups.

## Results

In total, 2,915 individuals were included in the three trials (Table 1). Most participants were male (70.9 %), and the overall mean age was 59.7 years (SD 9.3). Of the 2,667 subjects with end-point data, 973 (36.4 %) were found to have at least one conventional adenoma and 633 (23.7 %) had at least one serrated polyp during the follow-up period. Among individuals who had one or more serrated polyps during follow-up, 301 (47.6 %) also had one or more concurrent adenomas. Current smoking was associated with an increased risk of one or more adenomas (RR 1.29, 95 % CI 1.11–1.49) and former smoking with a slightly lower, but still significantly increased risk (RR 1.18, 95 % CI 1.05–1.32, *p*-trend for categories of smoking = 0.001, Table 2) relative to never smoking. Smoking intensity (number of cigarettes per day) did not show a significant trend (*p* = 0.17); however, duration of smoking was modestly associated with an increasing rising trend, as the highest two levels of exposure were associated with the largest risk of adenomas versus never smokers (25 to <35 years: RR 1.28, 95 % CI 1.09–1.49; and ≥35 years: RR 1.21, 95 % CI 1.05–1.41, *p*-trend = 0.006). Former smokers had a reduced risk of conventional adenomas compared to current smokers, particularly for those who quit between 1 and <20 years prior to study entry (RR 0.84, 95 % CI 0.73–0.98). RR estimates for advanced adenomas were similar to the overall RRs, though with wider CI compared to tubular adenomas, reflecting sample size differences (Table 2).

**Table 1:** Characteristics of the study populations in the Polyp Prevention Studies

Characteristics	APPS	CPPS	AFPPS	All trials
Participants, (*n*)	864	930	1,121	2,915
Participants with follow-up data, (*n*)	751	832	1,084	2,667
Duration (recruitment to end of treatment, mean ± SD), (years)	4.1 ± 0.3	4.0 ± 0.4	3.0 ± 0.5	3.6 ± 0.7
Age at randomization (mean ± SD), (years)	61.2 ± 8.3	61.0 ± 9.1	57.4 ± 9.6	59.7 + 9.3
Male sex, *n* (%)	684 (79.2)	672 (72.2)	712 (63.5)	2,068 (70.9)
Body mass index (mean ± SD) (kg/m)	26.9 ± 4.1	27.4 ± 4.4	27.4 ± 4.5	27.3 ± 4.3
Colon or colorectal cancer in first-degree relative, *n* (%)^b^	165 (19.5)	194 (23.9)	341 (37.3)	700 (24.0)
Treatment group, *n* (%)				
Placebo	214 (24.8)	466 (50.1)	169 (16.6)^a^	
Beta-carotene	217 (25.1)	–	–	
Beta-carotene, vitamin C, and vitamin E	208 (24.1)	–	–	
Vitamin C and vitamin E	214 (24.8)	–	–	
Calcium	–	464 (49.9)	–	
Folic acid	–	–	516 (50.5)	
Aspirin 81 mg	–	–	377 (33.6)	
Aspirin 325 mg	–	–	372 (33.2)	
Alcohol (mean ± SD), drinks per day	0.84 ± 1.61	0.60 ± 1.12	0.63 ± 1.06	0.68 ± 1.3
Smoking status				
Never	268 (31.7)	309 (33.2)	481 (43.0)	1,058 (36.6)
Former	389 (46.0)	442 (47.5)	473 (42.3)	1,304 (45.1)
Current	188 (22.3)	179 (19.3)	164 (14.7)	531 (18.4)
Pack-years				
None	268 (59.0)	309 (41.2)	481 (43.2)	1,058 (45.6)
0 to <30	22 (4.8)	205 (27.3)	319 (28.6)	546 (23.6)
30 to <75	90 (19.8)	163 (21.7)	253 (22.7)	506 (21.8)
≥75	74 (16.3)	73 (9.7)	61 (5.4)	208 (9.0)
Intensity (number of cigarettes/day)				
None	268 (32.2)	309 (33.4)	481 (43.0)	1,058 (36.8)
0 to <20	82 (9.8)	108 (11.7)	163 (14.6)	353 (12.3)
20 to <30	250 (30.0)	292 (31.5)	307 (27.5)	849 (29.5)
≥30	233 (28.0)	217 (23.4)	167 (14.9)	617 (21.5)
Duration (years)				
None	268 (32.5)	309 (41.1)	481 (43.1)	1,058 (39.3)
0 to <25	177 (21.5)	193 (25.7)	299 (26.8)	669 (24.8)
25 to <35	117 (14.2)	129 (17.2)	158 (14.2)	404 (15.0)
≥35	263 (31.9)	121 (16.1)	178 (15.9)	562 (20.9)
Time since quitting (years)				
None	205 (36.5)	189 (30.5)	176 (27.6)	570 (31.4)
1 to <20	238 (42.4)	284 (45.8)	262 (41.1)	784 (43.1)
≥20	118 (21.0)	147 (23.7)	199 (31.2)	464 (25.5)
Adenoma characteristics (at baseline)				
Number (Mean ± SD)	1.87 ± 1.34	1.88 ± 1.40	1.58 ± 0.98	1.9 ± 1.3
Advanced adenomas, *n* (%)	348 (40.3)	329 (19.6)	325 (29.0)	1,002 (34.4)
Right-sided location, *n* (%)	333 (38.5)	400 (23.8)	503 (44.9)	1,236 (42.4)

Counts do not necessarily add to the total sum due to missing data

^a^PPS3 used a 2 × 3 factorial design, and counts in this table for folic acid and aspirin are greater than the total in the study. Total number in the placebo group represents the number of individuals who did not receive either folic acid or aspirin 81/325 mg

^**b**^PPS1 and PPS2 collected family history with colon cancer; PPS3 specified colorectal cancer

**Table 2:** Association between cigarette smoking and risk of colorectal precursor lesions

	One or more adenomas		One or more advanced adenomas		One or more small tubular adenomas		One or more serrated polyps	
	*n* events	RR^a^ (95 % CI)	*n* events	RR^a^ (95 % CI)	*n* events	RR^a^ (95 % CI)	*n* events	RR^a^ (95 % CI)
Status								
Never	315	1.00 (ref)	76	1.00 (ref)	267	1.00 (ref)	178	1.00 (ref)
Former	472	1.18 (1.05, 1.32)	124	1.26 (0.95, 1.66)	396	1.15 (1.01, 1.32)	298	1.42 (1.20, 1.68)
Current	173	1.29 (1.11, 1.49)	41	1.29 (0.90, 1.86)	153	1.33 (1.12, 1.57)	152	2.01 (1.66, 2.44)
*p*-trend		0.001		0.18		0.001		<0.0001
Pack-years								
None	315	1.00 (ref)	76	1.00 (ref)	267	1.00 (ref)	178	1.00 (ref)
0 to <30	205	1.23 (1.07, 1.43)	56	1.44 (1.02, 2.03)	167	1.20 (1.02, 1.42)	125	1.30 (1.06, 1.60)
30 to <75	184	1.20 (1.04, 1.39)	48	1.29 (0.91, 1.83)	157	1.22 (1.03, 1.44)	153	1.84 (1.52, 2.22)
≥75	68	1.22 (1.00, 1.52)	18	1.22 (0.74, 2.00)	58	1.23 (0.97, 1.56)	56	1.96 (1.50, 2.56)
*p*-trend		0.09		0.56		0.11		<0.0001
Intensity (number of cigarettes/day)								
None	315	1.00 (ref)	76	1.00 (ref)	267	1.00 (ref)	178	1.00 (ref)
0 to <20	132	1.27 (1.08, 1.50)	33	1.28 (0.86, 1.89)	114	1.30 (1.08, 1.56)	67	1.17 (0.92, 1.50)
20 to <30	305	1.22 (1.07, 1.38)	76	1.25 (0.92, 1.70)	255	1.19 (1.03, 1.38)	234	1.80 (1.51, 2.13)
≥30	201	1.13 (0.98, 1.31)	52	1.23 (0.87, 1.75)	175	1.14 (0.97, 1.35)	145	1.57 (1.29, 1.92)
*p*-trend		0.17		0.30		0.25		<0.0001
Duration (years)								
None	315	1.00 (ref)	76	1.00 (ref)	267	1.00 (ref)	178	1.00 (ref)
0 to <*25*	237	1.17 (1.02, 1.34)	61	1.27 (0.92, 1.76)	198	1.14 (0.98, 1.34)	151	1.31 (1.09, 1.59)
25 to <35	155	1.28 (1.09, 1.49)	40	1.32 (0.92, 1.92)	131	1.27 (1.06, 1.51)	90	1.42 (1.14, 1.78)
≥35	195	1.21 (1.05, 1.40)	47	1.13 (0.79, 1.61)	170	1.23 (1.05, 1.46)	164	2.12 (1.75, 2.57)
*p*-trend		0.006		0.48		0.007		<0.0001
Time since quitting (years)								
None^b^	190	1.00 (ref)	47	1.00 (ref)	166	1.00 (ref)	169	1.00 (ref)
1 to <20	257	0.84 (0.73, 0.98)	61	0.77 (0.53, 1.11)	220	0.83 (0.70, 0.98)	175	0.69 (0.57, 0.82)
≥20	192	0.91 (0.77, 1.07)	55	0.95 (0.65, 1.41)	159	0.88 (0.73, 1.06)	101	0.60 (0.48, 0.75)
*p*-trend		0.26		0.82		0.16		<0.0001

^a^Risk ratios are adjusted for age, sex, clinical center, and trial

^b^Includes current smokers and individuals quitting smoking for <1 year

Current and former smoking was associated with an increased risk of one or more serrated polyps on follow-up colonoscopy (RR 2.01, 95 % CI 1.66–2.44; and RR 1.42, 95 % CI 1.20–1.68, respectively, *p*-trend <0.0001) (Table 2). There was a significant trend of increasing risk with greater levels of pack-years; compared to never smokers, those with >75 pack-year smoking history had a RR of serrated polyps of 1.96 (95 % CI 1.50–2.56, *p*-trend <0.0001). Both smoking intensity and duration also displayed trends of increasing risk with increasing exposure. For smokers of >35 years, the RR of serrated polyps was 2.12 (95 % CI 1.75–2.57). In addition, smoking cessation was clearly associated with reduced risk in comparison with continued smoking (RR 0.60; 95 % CI 0.48–0.75) for those quitting 20 or more years ago compared to current smokers. When we examined the association by size, we observed a slightly lower estimated relative risk of serrated lesions greater than 1 cm (current: RR 1.62, 95 % CI 1.27–2.07 and former: RR 1.21, 95 % CI 0.98–1.49).

Smoking-associated risks of different polyp types by location in the colorectum are shown in Table 3. For conventional adenomas, risks associated with current smoking appeared slightly higher in the left colorectum (RR 1.50; 95 % CI 1.24–1.83) compared to the right colon (RR 1.27, 95 % CI 1.04–1.55). Risk estimates for pack-years, intensity, and duration were similarly higher in the left colorectum versus right colon.

**Table 3:** Association between smoking status, pack-years, and duration and risk of precursor lesions by location in the colorectum

	Adenomas				Serrated polyps			
	Right colon		Left colorectum		Right colon		Left colorectum	
	*n* events	RR^a^ (95 % CI)	*n* events	RR^a^ 95 % CI	*n* events	RR^a^ 95 % CI	*n* events	RR^a^ 95 % CI
Status								
Never	211	1.00 (ref)	200	1.00 (ref)	81	1.00 (ref)	146	1.00 (ref)
Former	296	1.09 (0.93, 1.27)	333	1.30 (1.14, 1.52)	108	1.11 (0.84, 1.47)	262	1.53 (1.27, 1.83)
Current	116	1.27 (1.04, 1.55)	128	1.50 (1.24, 1.83)	37	1.09 (0.75, 1.58)	140	2.28 (1.85, 2.80)
* p*-trend		0.02		<0.0001		0.67		<0.0001
Pack-years								
None	211	1.00 (ref)	200	1.00 (ref)	81	1.00 (ref)	146	1.00 (ref)
0 to <30	134	1.22 (1.00, 1.49)	133	1.28 (1.05, 1.56)	46	1.05 (0.73, 1.49)	106	1.34 (1.07, 1.68)
30 to <75	108	1.05 (0.85, 1.29)	141	1.47 (1.21, 1.77)	40	1.06 (0.73, 1.53)	139	2.05 (1.66, 2.52)
≥75	48	1.26 (0.96, 1.67)	50	1.42 (1.11, 1.87)	16	1.20 (0.71, 2.03)	53	2.25 (1.68, 2.99)
* p*-trend		0.24		0.009		0.51		<0.0001
Intensity (number of cigarettes per day)								
None	211	1.00 (ref)	200	1.00 (ref)	81	1.00 (ref)	146	1.00 (ref)
0 to <20	87	1.29 (1.04, 1.61)	82	1.24 (0.99, 1.55)	26	0.97 (0.63, 1.48)	59	1.27 (0.97, 1.67)
20 to <30	189	1.11 (0.93, 1.32)	230	1.45 (1.23, 1.71)	72	1.19 (0.87, 1.61)	207	1.95 (1.61, 2.36)
≥30	132	1.07 (0.88, 1.30)	143	1.28 (1.05, 1.55)	44	1.04 (0.72, 1.50)	132	1.73 (1.39, 2.15)
* p*-trend		0.91		0.005		0.60		<0.0001
Duration (years)								
None	211	1.00 (ref)	200	1.00 (ref)	81	1.00 (ref)	146	1.00 (ref)
0 to <25	151	1.10 (0.92, 1.33)	167	1.28 (1.07, 1.54)	58	1.11 (0.80, 1.54)	129	1.37 (1.11, 1.69)
25 to <35	94	1.13 (0.91, 1.40)	110	1.42 (1.16, 1.74)	27	0.90 (0.59, 1.38)	85	1.64 (1.29, 2.08)
≥35	128	1.19 (0.97, 1.44)	141	1.37 (1.13, 1.66)	45	1.19 (0.83, 1.71)	148	2.36 (1.91, 2.92)
* p*-trend		0.10		0.001		0.59		<0.0001
Time since quitting (years)								
None^b^	129	1.00 (ref)	141	1.00 (ref)	40	1.00 (ref)	155	1.00 (ref)
1 to <20	166	0.80 (0.66, 0.98)	178	0.79 (0.66, 0.96)	62	0.99 (0.68, 1.46)	158	0.67 (0.56, 0.82)
≥20	113	0.82 (0.65, 1.04)	138	0.89 (0.72, 1.10)	41	0.95 (0.61, 1.47)	85	0.54 (0.43, 0.69)
* p*-trend		0.10		0.26		0.82		<0.0001

^a^Risk ratios are adjusted for age, sex, clinical center, and trial

^b^Includes current smokers and individuals quitting smoking for <1 year

A more marked pattern was evident for serrated polyps. In comparison with never smokers, current smoking was associated with a RR of 2.28 (95 % CI 1.85–2.80) for serrated polyps in the left colorectum, but no increased risk was seen on the right (RR 1.09; 95 % CI 0.75–1.58). As with conventional adenomas, similar patterns were observed for pack-years, intensity, and duration.

Associations between smoking and occurrence of serrated polyps alone were more pronounced (for current smoking vs. never smoking: RR 2.33, 95 % CI 1.76–3.07, Table 4). For the association between smoking and the combination of serrated polyps and adenomas, risk estimates were similar to those of serrated polyps only (for current smoking vs. never smoking: RR 2.27, 95 % CI 1.68–3.06). This pattern was consistently seen for pack-years, smoking intensity and duration, and time since quitting. When we stratified by sex, estimates of risk of smoking status were similar between men and women (Table 5), though with smaller numbers results were not statistically significant in women. Results for smoking exposures defined by pack-years, intensity, and timing also showed similar findings between men and women (data not shown). No significant differences in the estimates of the association between smoking variables and risk by treatment group.

**Table 4:** Association between smoking and risk of adenomas, serrated polyps, and concurrent adenomas and serrated polyps

	Only adenomas		Only serrated polyps		Adenomas and serrated polyps	
	*n* events	RR^a^ (95 % CI)	*n* events	RR^a^ (95 % CI)	*n* events	RR^a^ (95 % CI)
Status						
Never	236	1.00 (ref)	91	1.00 (ref)	79	1.00 (ref)
Former	318	1.15 (1.00, 1.33)	128	1.40 (1.09, 1.79)	154	1.66 (1.29, 2.13)
Current	107	1.31 (1.08, 1.58)	76	2.33 (1.76, 3.07)	66	2.27 (1.68, 3.06)
* p*-trend		0.007		<0.0001		<0.0001
Pack-years						
None	236	1.00 (ref)	91	1.00 (ref)	79	1.00 (ref)
0 to <30	140	1.20 (1.00, 1.44)	52	1.19 (0.87, 1.61)	65	1.65 (1.22, 2.23)
30 to <75	105	1.12 (0.92, 1.36)	65	1.76 (1.32, 2.34)	79	2.12 (1.59, 2.82)
≥75	44	1.28 (0.97, 1.67)	28	2.49 (1.67, 3.71)	24	2.00 (1.31, 3.05)
* p*-trend		0.14		<0.0001		0.001
Intensity (number of cigarettes/day)						
None	236	1.00 (ref)	91	1.00 (ref)	79	1.00 (ref)
0 to <20	100	1.30 (1.07, 1.58)	30	1.16 (0.79, 1.68)	32	1.47 (1.01, 2.12)
20 to <30	184	1.16 (0.98, 1.36)	101	1.80 (1.40, 2.34)	121	2.05 (1.58, 2.64)
≥30	136	1.14 (0.95, 1.37)	71	1.79 (1.33, 2.40)	65	1.65 (1.21, 2.24)
* p*-trend		0.38	<0.0001			0.0004
Duration (years)						
None	236	1.00 (ref)	91	1.00 (ref)	79	1.00 (ref)
0 to <*25*	161	1.15 (0.97, 1.36)	69	1.26 (0.96, 1.70)	76	1.60 (1.20, 2.14)
25 to <35	111	1.31 (1.09, 1.59)	42	1.62 (1.16, 2.27)	44	1.70 (1.22, 2.39)
≥35	113	1.10 (0.92, 1.34)	68	2.05 (1.52, 2.79)	82	2.18 (1.63, 2.91)
* p*-trend		0.15		<0.0001		<0.0001
Time since quitting (years)						
None^b^	116	1.00 (ref)	82	1.00 (ref)	74	1.00 (ref)
1 to <20	171	0.81 (0.67, 0.98)	78	0.58 (0.44, 0.77)	86	0.65 (0.49, 0.86)
≥20	134	0.89 (0.72, 1.11)	41	0.53 (0.37, 0.75)	58	0.70 (0.51, 0.96)
* p*-trend		0.30		0.0003		0.03

^a^Risk ratios are adjusted for age, sex, clinical center, and trial

^b^Includes current smokers and individuals quitting smoking for <1 year

**Table 5:** Association between smoking status and risk of adenomas, serrated polyps, and concurrent adenomas and serrated polyps stratified by sex

	Only adenomas		Only serrated polyps		Adenomas and serrated polyps	
	*n* events	RR^a^ (95 % CI)	*n* events	RR^a^ (95 % CI)	*n* events	RR^a^ (95 % CI)
Men						
Never	207	1.00 (ref)	106	1.00 (ref)	53	1.00 (ref)
Former	387	1.19 (1.04, 1.35)	233	1.42 (1.16, 1.73)	124	1.48 (1.09, 2.00)
Current	137	1.34 (1.13, 1.58)	103	1.90 (1.50, 2.41)	53	2.03 (1.41, 2.90)
* p*-trend		0.0009		<0.0001		0.0001
Women						
Never	108	1.00 (ref)	72	1.00 (ref)	26	1.00 (ref)
Former	85	1.18 (0.93, 1.50)	65	1.41 (1.05, 1.89)	30	1.86 (1.13, 3.06)
Current	36	1.16 (0.84, 1.60)	49	2.22 (1.61, 3.05)	13	1.68 (0.89, 3.16)
* p*-trend		0.36		<0.0001		0.12

^a^Risk ratios are adjusted for age, sex, clinical center, and trial

## Discussion

In this combined analysis of three closely followed cohorts participating in adenoma prevention trials, we found strong evidence that cigarette smoking is associated with the development of serrated polyps in the colorectum. In contrast, we found only weak associations between smoking and risk of conventional adenomas (tubular or advanced), and no substantive differences by sex. For both serrated polyps and adenomas, risks associated with smoking were stronger in the left colorectum compared to the right colon. The association with smoking was very similar for participants with serrated polyps only and those with both serrated polyps and adenomas. In examining trends with duration, pack-years, and intensity, higher levels of exposure were consistently associated with higher risk of serrated polyps. Such patterns were not observed consistently for conventional adenomas.

A few other studies have reported a strong link between smoking and serrated polyps, with point estimates of risk notably higher than for adenomas [6, 10, 11]. Some data suggest that smoking appears to be an important risk factor for SSA/Ps [26–28]. However, we, and other investigators [20], observed no association between smoking and incident serrated polyps in the right colon, where SSA/Ps comprise a significant proportion of serrated polyps. Consistent with our data and others [10–15], the highest smoking-associated risk of serrated lesions is in the left colorectum. The lack of association with right-sided serrated lesions, and the weaker association with large serrated lesions, would suggest that smoking either does not play a significant role in initiation of precursors that give rise to the development of proximal CIMP tumors (which includes most sporadic MSI carcinomas), or that smoking acts at a later stage in the serrated pathway carcinogenic process (i.e., promoting the transition from advanced serrated polyp to MSI cancer). Alternatively, smoking may be linked to colorectal carcinogenesis by effects on the “traditional serrated pathway” via TSA precursor lesions that are typically located in the distal colorectum [29].

The controversy regarding whether or not smoking is associated with CRC [2–4] may be at least in part explained by differences in the two molecular pathways to carcinogenesis. In detailed analyses examining molecular classification of tumors, smoking has been shown to be associated only with a subset characterized by somatic *BRAF* V600E mutation [30–32], KRAS wild type [33], CIMP [32, 34], and MSI [30, 35, 36], all distinguishing molecular and epigenetic characteristics of the serrated pathway. Smoking is also more strongly associated with proximal versus distal CRC [33, 37, 38]. These data show that smoking acts on the serrated pathway at some point. Our null findings for an association between smoking and serrated lesions in the right colon suggest that this would occur with the conversion of SSA/Ps to carcinoma, potentially via promotion of methylation of MLH1 [39], and therefore, the role of smoking in the initiation phases of polyp development is uncertain. However, compounds in cigarette smoke have been shown to alter DNA methylation patterns in a number of cancer-related genes and genome-wide methylation studies [40]. Furthermore, in a large pooled analysis examining the effect of timing of exposure, smoking cessation (compared to continued smoking) conferred a markedly reduced risk of cancers in right colon, but not in the left colon [4], supporting the idea that smoking may be a key in promoting late pathway progression to serrated carcinomas.

From our prospective clinical trials, we also observed overall estimates of association between smoking and risk of adenomas were weaker (relative risks of 1.2 for former smoking and 1.3 for current smoking) compared to those reported in a previous meta-analysis (relative risks of 1.5 for former smoking and 2.0 for current smoking) [5]. One possible explanation for the difference is that our trial considered incident adenomas in individuals who had all polyps removed at baseline colonoscopies 3–5 years prior, while the previous studies investigated prevalent lesions. If smoking has different effects on initiation versus persistence of adenomas, this could explain the lower risk estimates observed in our study. Furthermore, it is well known that ORs overestimate RR, especially when outcomes are not rare [41].

A few case–control studies [6, 10, 19] suggest that the smoking-associated risk of concurrent adenomas and serrated polyps is even higher than the estimates for the occurrence of only serrated polyps. In these studies, the observed estimates of the OR for current smoking compared to never smoking for both adenomas and serrated lesions ranged from 6.1 to 6.2; the OR estimates for serrated ranged from 4.1 to 4.4 and for adenomas only from 1.3 to 2.0 (Table 6). One additional case–control study by Burnett-Hartman et al. [20] did not observe an elevated risk of concurrent adenomas and serrated polyps versus serrated only, in agreement with our finding. It is not clear why our findings and those of Burnett-Hartman et al. [20] do not agree with earlier publications [6, 10, 19], but in the absence of a definite “separate phenotype” of concurrent adenomas and serrated polyps associated with smoking, the observed association between smoking and conventional adenomas cannot be explained by the inclusion of persons who also have serrated polyps. Smoking must have some effects on adenomas and the chromosomal instability pathway as well.

**Table 6:** Summary of epidemiologic studies of smoking and adenomas, serrated polyps, and both types

References	Population, location	Study design	Adenomas only Category Category *n* OR/RR (95 % CI) current versus never smokers	Serrated polyps only Category Category *n* OR/RR (95 % CI) current versus never smokers	Adenomas and serrated polyps Category *n* OR/RR (95 % CI) current versus never smokers
Figueiredo 2014 [current study]	Multicenter, PPSG	RCT/cohort, colonoscopy-based	*n* = 661 RR = 1.31 (1.08, 1.58)	*n* = 295 RR = 2.33 (1.76, 3.07)	*n* = 299 RR = 2.27 (1.68, 3.06)
Burnett-Hartman [20]	Group Health Cooperative, Washington State	Case–control, colonoscopy-based	*n* = 628 OR = 1.56 (0.99, 2.44)	*n* = 594 OR = 3.00 (1.93, 4.66)	*n* = 247 OR = 2.82 (1.65, 4.81)
Fu [19]	Vanderbilt Medical Center and Tennessee Valley VA, Nashville, TN	Case–control, colonoscopy-based	*n* = 1,444 OR = 1.96 (1.61, 2.38)	*n* = 662 OR = 4.44 (3.47, 5.67)	*n* = 437 OR = 6.10 (4.51, 8.25)
Ji [6]	Multicenter, PLCO	Case–control, sigmoidoscopy-based	*n* = 2,500 OR = 1.8 (1.5, 2.1)	*n* = 1,762 OR = 4.4 (3.7, 5.2)	*n* = 582 OR = 6.2 (4.7, 8.3)
Morimoto [10]	Multiclinic private gastroenterology practice, Minneapolis, MN	Case–control, colonoscopy-based	*n* = 437 OR = 1.3 (0.8, 2.3)	*n* = 219 OR = 4.1 (2.2, 7.6)	*n* = 138 OR = 6.1 (2.8, 13.5)

*RR* risk ratio; *OR* odds ratio; *CI* confidence interval; *RCT* randomized controlled trial; *PPSG* Polyp Prevention Study Group; *PLCO* prostate, lung, colorectal, and ovarian cancer screening trial

We did not observe material differences in smoking-associated risks with the size of adenomas or advanced histological features (both components of the advanced adenoma category) or significant heterogeneity by gender, in agreement with some, but not all studies [5, 6, 12]. This may be related to limited sample size when stratifying by gender and polyp characteristics, especially given adenomas are more common in males and the infrequency of advanced adenomas in the pooled studies.

Our study has some limitations and notable strengths. The generalizability of our results may be affected by the fact that all participants in this clinical trial were volunteers who had a previous history of at least one colorectal adenoma, so they represent a restricted part of the colon cancer risk spectrum. Despite expanding upon our earlier study [8] with pooled data across three clinical trials, we had a limited sample size to investigate risk of some of the specific histologic subtypes, in particular after stratification by other patient characteristics such as gender. Furthermore, because our studies were conducted before the current understanding of the serrated pathway had been developed, we were unable to subclassify serrated polyps into more meaningful outcome categories. Moreover, since proximal serrated polyp detection by endoscopy is often incomplete [42, 43], it is possible that some participants had undetected proximal serrated lesions, which may have biased our results toward the null for analyses of serrated lesions in particular. We focus here on modeling smoking patterns. Although the pack-years variable could be influenced by intensity in nonadditive terms [44], our results were consistent across multiple smoking variables, contributing to internal validity. The strengths of the current study include pooled analysis of individual-level data from large studies with colonoscopy-based outcome assessment, long follow-up, and systematic collection of patient characteristics in a uniform fashion by the Polyp Prevention Study Group.

In summary, we have found that smoking is associated more strongly with serrated polyps than conventional adenomas and that there is not a “separate phenotype” of smokers with both adenomas and serrated polyps. Multiple lines of evidence now indicate that cigarette smoking is associated both with distal serrated polyps (which are thought to have a lower potential for malignancy) as well as with MSI-H colorectal cancer, which is largely proximal. This apparent paradox could be explained by smoking having multiple effects on serrated pathway carcinogenesis, including initiation of left-sided serrated polyp, as well as a role in later stages of progression from advanced proximal serrated polyps to carcinoma.
